# COVID-19 and the law in Uganda: a case study on development and application of the public health act from 2020 to 2021

**DOI:** 10.1186/s12889-023-15555-5

**Published:** 2023-04-25

**Authors:** Martha Isabella Achan, Immaculate Nabukenya, Sarah Mitanda, Joanita Nakacwa, Herbert Bakiika, Maureen Nabatanzi, Justine Bukirwa, Aisha Nakanwagi, Lydia Nakiire, Cedric Aperce, Aaron Schwid, Solome Okware, Ekwaro A. Obuku, Mohammed Lamorde, Brian Luswata, Issa Makumbi, Allan Muruta, Henry G. Mwebesa, Jane Ruth Aceng Ocero

**Affiliations:** 1 Infectious Diseases Institute, Makerere University, P.O. Box 22418, Kampala, Uganda; 2 Ministry of Justice and Constitutional Affairs, P.O Box 7183, Kampala, Uganda; 3 Resolve to Save Lives, New York 100 Broadway, 4th Floor, New York, NY 10005 USA; 4 Vital Strategies, New York 100 Broadway, 4th Floor, New York, NY 10005 USA; 5 World Health Organization, Uganda Country Office, P. O. Box 24578, Kampala, Uganda; 6 Ministry of Health, P.O. Box 7272, Kampala, Uganda

**Keywords:** Public Health Law, Uganda, COVID-19, Pandemic, Rules, Non-pharmaceutical interventions

## Abstract

**Background:**

Despite the discovery of vaccines, the control, and prevention of Coronavirus disease 2019 (COVID-19) relied on non-pharmaceutical interventions (NPIs). This article describes the development and application of the Public Health Act to implement NPIs for COVID-19 pandemic control in Uganda.

**Methods:**

This is a case study of Uganda’s experience with enacting COVID-19 Rules under the Public Health Act Cap. 281. The study assessed how and what Rules were developed, their influence on the outbreak progress, and litigation. The data sources reviewed were applicable laws and policies, Presidential speeches, Cabinet resolutions, statutory instruments, COVID-19 situation reports, and the registry of court cases that contributed to a triangulated analysis.

**Results:**

Uganda applied four COVID-19 broad Rules for the period March 2020 to October 2021. The Minister of Health enacted the Rules, which response teams, enforcement agencies, and the general population followed. The Presidential speeches, their expiry period and progress of the pandemic curve led to amendment of the Rules twenty one (21) times. The Uganda Peoples Defense Forces Act No. 7 of 2005, the Public Finance Management Act No. 3 of 2015, and the National Policy for Disaster Preparedness and Management supplemented the enacted COVID-19 Rules. However, these Rules attracted specific litigation due to perceived infringement on certain human rights provisions.

**Conclusions:**

Countries can enact supportive legislation within the course of an outbreak. The balance of enforcing public health interventions and human rights infringements is an important consideration in future. We recommend public sensitization about legislative provisions and reforms to guide public health responses in future outbreaks or pandemics.

## Background

The novel Severe Acute Respiratory Coronavirus (SARS-COV-2) causing the Coronavirus Disease (COVID-19) outbreak was reported in Wuhan, Hubei China in December 2019 [1]. On 30th January 2020, the World Health Organization (WHO) declared the COVID-19 outbreak a Public Health Event of International Concern [2], and a pandemic on 11th March 2020 [3]. Further, the first case in Africa was documented in Egypt on 14th February 2020, whilst in Uganda, it was on 20th March 2020 at Entebbe International Airport. Due to the extreme ability of the virus to spread widely and rapidly, several countries including Canada, United States of America, Northern Ireland used domestic and international laws to respond to the outbreak [4–6].

Uganda is signatory to the International Health Regulations (IHR) (2005) [7] implemented through the Integrated Disease Surveillance and Response (IDSR) guidelines [8]. These require core capacities to prevent, detect and respond to public health emergencies, such as COVID-19. In October 2019, the Ministry of Health and the Ministry of Justice & Constitutional Affairs) accelerated amendments to the Public Health Act [9] to domesticate the IHR (2005) and incorporate the IDSR guidelines [10].

The Public Health Act is the governing law on public health in Uganda and is implemented by the Ministry of Health [9]. Consequently, the Public Health Act, in Part V assigns the role of prevention and control of outbreaks and pandemics to the central government. Likewise, Sect. 5 authorizes local governments to enforce all provisions to ensure the promotion of public health. The Public Health Act was enacted in 1935 during the British colonial rule and has never been substantially amended, except re-codified two times. In 1964, following Uganda’s Independence, it was re-codified to the Public Health Act Chap. 269 laws of Uganda. During this period, Uganda applied it to respond to a Yellow Fever outbreak in Bundibugyo, and Plague outbreak in Iganga and Kasese Districts. The Act was again re-codified in 2000 to the Public Health Act, Chap. 281. It had not been applied to respond to any disease outbreak until the COVID-19 outbreak. The Act is similar to public health laws of other African countries in the Commonwealth (Kenya’s Public Health Act, Chap. 242; South Africa’s Public Health Act, 36 of 1919; Zambia’s Public Health Act, Chap. 295; Malawi’s Public Health Act, Chap. 34:01; Botswana’s Public Health Act, Chap. 63:01 and Zambia Public Health Act, Chap. 295).

Uganda mainly applied the Public Health Act Chap. 281 [9] of the laws of Uganda to respond to COVID-19, particularly for the Non-Pharmaceutical Interventions (NPIs) [11]. Other supporting laws included Article 189 (Sixth Schedule) of the 1995 Constitution of Uganda [12], which outlines the government’s responsibility for health policy, control, and management of outbreaks and disasters. Equally, Sect. 179 of the Local Government Act Chap. 243 (second schedule) [13] authorizes Local Governments to prevent and control diseases outbreaks. Also, the Public Finance Management Act of 2015 in Sect. 25 provides for application for supplementary budgets to respond to public health emergencies [14]. Another law that is applied to respond to public health emergencies is the Uganda Peoples’ Defence Forces Act 2005 [15].The national policy for Disaster Preparedness and Management 2010 [16] is the key instrument that guides the government in responding to public health emergencies in the form of disasters.

Although Uganda had not completed this amendment of the Public Health Act, the COVID-19 pandemic demanded legislation to legally implement control measures. Sections 10, 11, 27, and 36 of the Public Health Act required that the Minister of Health implements the health measures listed therein by enacting subsidiary legislation or Rules. The Public Health Act gives the Minister of Health the power to make Rules and Orders under Sects. 11, 27, 29, and 36 respectively. These Rules are recognized as law under Sect. 14 of the Interpretation Act and form part of the legal framework of Uganda [17]. To prepare and respond to the COVID-19 pandemic, the Minister made several Rules and Orders by publication in the *Government Gazette*.

There is a scarcity of information on the process undertaken to develop the COVID-19 Rules and their subsequent application in response to control and prevention of the COVID-19 pandemic and future public health emergencies in Uganda. This case study documents Uganda’s process of development, application, and experience with amending the Public Health Act Rules that guided COVID-19 control. The information in this paper can be useful to Uganda and other countries seeking to understand the legal frameworks applicable when responding to future disease outbreaks.

## Methods

This case study documents Uganda’s experience in applying the Public Health Act and attendant Rules when responding to COVID-19 outbreak. A ‘case’ was defined as the ‘process of changing the Rules’ after a presidential speech containing health measures requiring public action [18]. In this study Non-Pharmaceutical Interventions (NPIs) means disease preventive public health measures such as hand washing, social distancing, wearing masks and others that do not involve use of vaccines or medicines [19]. In addition, this study assessed how the Rules, other supporting laws, and policies were applied from March 2020 to October 2021. The assessments of interest in this study were legal: priority NPIs, Rules signed, lawsuits heard, and court decisions made.

## Context of the legal regime in Uganda

During the study period March 2020 to 30 October 2021, Uganda experienced two peaks (waves) of COVID-19, with 126,236 cases and 3,215 deaths [20]. Notification, suppression, and prevention of infectious diseases in Uganda are implemented using subsidiary laws made under the Public Health Act. Uganda is part of the Commonwealth, hence applying Common Law. The country gained its independence from Great Britain in 1962 and currently, the 1995 Constitution [12] is the supreme law. Sections 14 and 15 of the Judicature Act state that the applicable laws are written laws or statutory laws, customary laws, common law, and doctrines of equity [21]. There are three arms of government that is the Executive (the President and the Cabinet of Ministers), Parliament or legislature, and Judiciary [12]. All the arms of Government participate in making laws. The Executive and Parliament approve the principal laws or Acts of Parliament. The responsible Cabinet Minister makes the subsidiary laws or statutory instruments for example Rules. The Judiciary makes the case law through judgements of court cases.

## The process of preparing the rules and orders

**Fig. 1:**
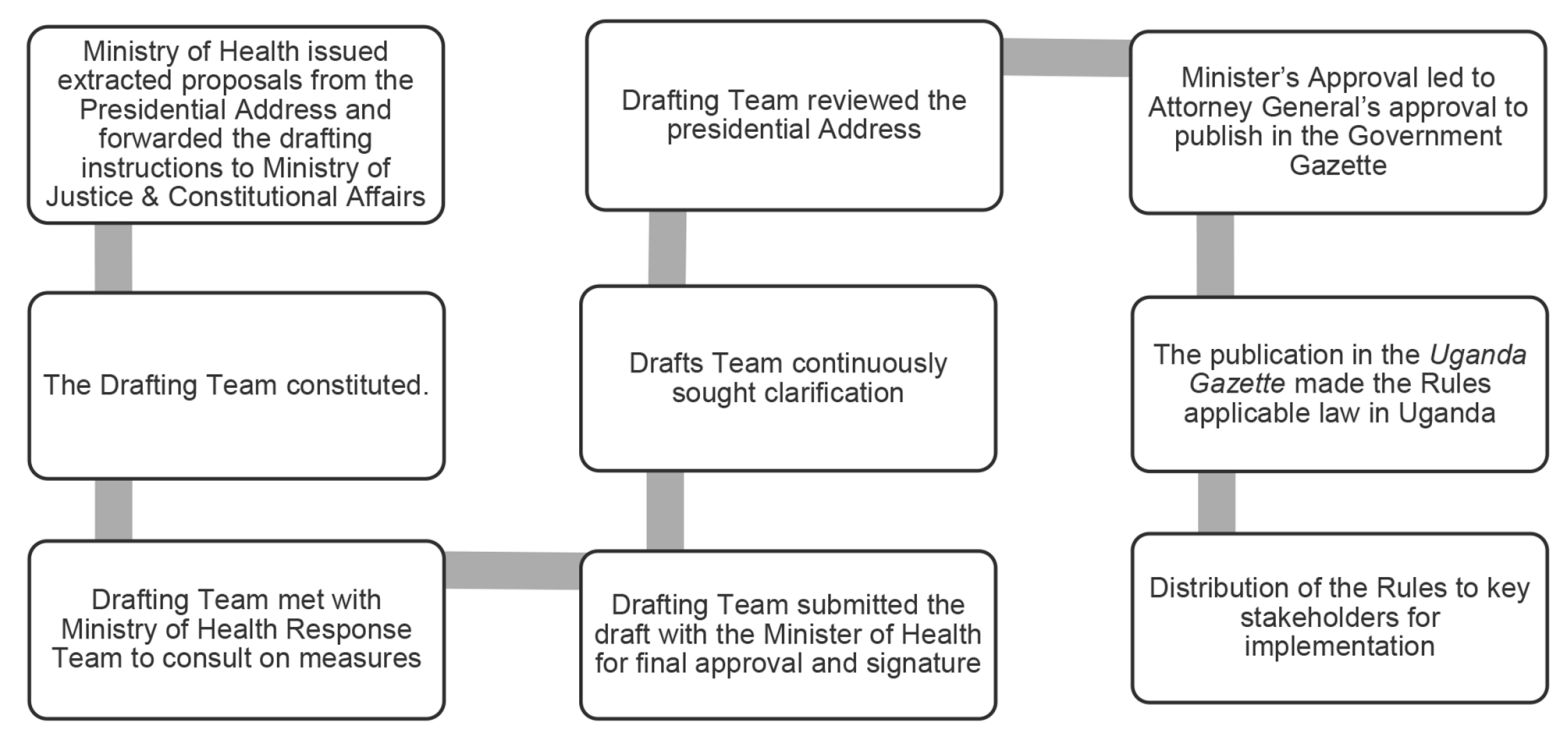
Flow chart showing the process for making each of the COVID-19 Rules. A process map showing the general methodology followed to make the Rules and Orders. Moving from left to right, we show the steps followed in Uganda to expound the respective section of the Act into an Order or Rule based on the guidance by the Minister of Health

The process of preparing Rules and Orders is in the Public Service Standing Orders, 2010, under the Public Service Act No. 9 of 2008. Additionally, paragraph (Q-b) in the Public Service Standing Orders, provides the process for making statutory instruments. Once the President provided guidance on control measures [22], the Ministry of Health prepared the proposals like closing schools, closing places of worship, restricting entry into the country, and so on. The Minister of Health forwarded drafting instructions to the Directorate of the First Parliamentary Counsel at Ministry of Justice and Constitutional Affairs. The Drafting Team comprised of the Ministry of Justice and Constitutional Affairs and the Ministry of Health. The team reviewed the enabling provisions in the Public Health Act and other related laws in Uganda. Following this, the Drafting Team met with the Ministry of Health outbreak response team to consult on the examination, quarantine, admission/isolation of COVID-19 patients, and other control options before the final drafting. The Drafting Team also reviewed the Presidential Address for policy guidance on the response to the COVID-19 pandemic. However, the Drafting Team continuously sought clarification on from the Ministry of Health on some of the health measures in the Presidential Address for example the difference between arcades and shopping malls; the age limit for going to church, and the health restrictions for open-air versus in-door places of worship. Subsequently, the Drafting Team shared the Rules with the Minister of Health for approval. This led to the Attorney General’s approval to publish the Rules in the *Government Gazette*, and upon publication, the Rules become applicable law (Fig. 1).

## Document review

The study reviewed primary data sources such as the Presidential Addresses on COVID-19 measures [22], Ministerial letters, Public Health Act [9], Uganda Peoples Defence Forces Act (UPDF Act) [15], Disaster Management Policy [16], Public Health Rules, health and security operational guidelines, rules issued by other public authorities. Court cases related to health and health emergencies during the COVID-19 pandemic were also reviewed. These documents were accessed and analyzed for content manually by the lead author (MIA), an Advocate of the High Court in Uganda. These sources were public repositories, specifically the open access data base of the Uganda Legal Information Institute (https://ulii.org/) for standing laws of Uganda, websites of the State House (https://statehouse.go.ug/), Uganda Media Centre (https://www.mediacentre.go.ug/presidents-statements), for Presidential speeches and pronouncements; and the Government of Uganda COVID-19 Response Information Hub (https://covid19.gou.go.ug/), the World Health Organization dashboard for COVID-19 cases.

## Results

### Presidential speeches on COVID-19

The President addressed the nation on several occasions throughout the COVID-19 outbreak [22]. The presidential speeches included status updates on the national response to the COVID-19 outbreak; health guidance and NPIs. Some of the speeches were further explanation on the directives that were not clear. All in all, these speeches pronounced 35 public health measures (NPIs) to control the spread of COVID-19. The 21 Rules and Orders resulted from these public health measures. These included the controlled movement of vehicles, vessels, and aircraft; closure of international borders (except for cargo); closure of education facilities; closure of places of prayers; curfew, and mandatory wearing of facial masks (Table 1).

**Table 1:** List of the Rules developed and used to control COVID-19 in Uganda, 2020–2021

Sections of the Act, Rules and Orders (n = 4)	Title of the Rules and Orders (n = 22)	Notes
Minister’s power to notify – Sect. 10	1) Public Health (Notification of COVID-19) Order, No. 45 of 2020	The Order declared COVID-19 a notifiable disease.
Control of COVID-19 – Sects. 11 and 27	1) Public Health (Control of COVID-19) Rules, No. 52 of 2020 2) Public Health (Control of COVID-19) (No.2), Rules, No. 55 of 2020 3) Public Health (Control of COVID-19) (Amendment) Rules, No. 57 of 2020 4) Public Health (Control of COVID-19) (No. 2) (Amendment) Rules, No. 58 of 2020 5) Public Health (Control of COVID-19) (Amendment) (No. 2) Rules, No. 63 of 2020 6) Public Health (Control of COVID-19) (No. 2) (Amendment No. 2) Rules, No. 64 of 2020 7) Public Health (Control of COVID-19) (Amendment No. 4) Rules, No. 79 of 2020 8) Public Health (Control of COVID − 19) (No. 2) (Amendment No. 4) Rules, No. 80 of 2020 9) Public Health (Control of COVID-19) Rules, No. 83 of 2020 10) Public Health (Control of COVID-19) (Amendment) Rules, No. 94 of 2020 11) Public Health (Control of COVID-19) (Amendment No. 2) Rules, No. 112 of 2020 12) Public Health (Control of COVID-19) Rules, 2021, No. 38 of 2021 13) Public Health (Control of COVID-1919) (Amendment No.1) Rules, No. 59 of 2021	These Rules were made after the declaration of COVID-19 a notifiable disease. These were Rules governing the Non-Pharmaceutical interventions (NPIs) i.e. community quarantine including closure of congregate settings such as schools, places of worship, markets, shopping malls, arcades, bars and cinemas. Additionally, marriage ceremonies, vigils, funerals were restricted, and the sports events or group exercising were specifically prohibited. These Rules provided for exemptions for “essential services and workers”, for example medics, legal and insurance. The amendment of the Rules was based on levels of the outbreaks, for example, re-opening of schools and closing them.
Powers to enforce precautions at borders – Sect. 36	1) Public Health (Prevention of COVID-19) (Requirements and Conditions of Entry into Uganda) Order, No. 46 of 2020 2) Public Health (Requirements and Conditions of Entry into Uganda) (Amendment) Order, No. 37 of 2021	These Order guided testing, medical examination, quarantine and other infection prevention and control measures at border points: land, water and airports.
Prohibition of entry into Uganda – Sect. 36	1) Public Health (Prohibition of Entry into Uganda) Order, No. 53 of 2020 2) Public Health (Prohibition of Entry into Uganda) (Amendment) Order, No. 56 of 2020 3) Public Health (Prohibition of Entry into Uganda) (Amendment No. 2) Order, No. 65 of 2020 4) Public Health (Prohibition of Entry into Uganda) (Amendment No. 4) Order, No. 81 of 2020 5) Public Health (Prohibition of Entry into Uganda) Order, No. 84 of 2020 6) Public Health (Prohibition of Entry into Uganda) (Revocation) Order, No. 113 of 2020	These Rules generally closed Uganda’s borders, restricted the number of persons in cargo vessels, vehicles or aircrafts, restricted entry of persons infected with COVID-19 and administered their isolation/quarantine, treatment and care.

Contains information on the salient features of the COVID-19 Rules and Orders. In the first column, we show the sections in the Public Health Act where the respective Rules and Orders align. In the second column, we present the citation of the Rules and Orders in sequence of publication in The Uganda Gazette. The notes in the third column give a description of main provisions in the Rules and Orders.

### Declaration of COVID-19 as a notifiable disease

Being new, COVID-19 was not among the notifiable diseases in the Public Health (Notifiable Diseases) Order Statutory Instrument 281–22. The Statutory Order is made under Sect. 10 (power to declare notifiable disease) the Public Health Act [23]. On 17th March 2020, the Minister of Health made the Public Health (Notification of COVID-19) Order, No. 45 of 2020 which declared COVID-19 a notifiable disease. Order 3 provided for Sect. 11 (Power to make Rules), Part IV (prevention and suppression of infectious diseases), and Sect. 36 (Power to Enforce Precautions at Borders) of the PHA that applied to control of COVID-19. Section 11 thus placed the responsibility of reporting (notifying) on the heads of a family; employers; heads of schools and local authorities. These persons were supposed to report any suspected case of COVID-19 to the medical officers or to the medical practitioners.

### Control of COVID-19 rules

Following the declaration of COVID-19 as a notifiable disease, Sects. 11, 27, and 36 of the Public Health Act became applicable to the response. On 24th March 2020, the Rules enforcing Sects. 11 and 27 were published as Public Health (Control of COVID-19) Rules [11]. Depending on the level of the COVID-19 outbreak, the Minister amended the Rules 12 times. Examples include the Rules on opening and closing of schools; closing and opening places of prayers; imposition of curfew; enforcing lockdown, and restrictions on the movement of passenger motorcycles.

### Health measures at the points of Entry

On 17th March 2020, an Order to control entry into Uganda was made under Sect. 36 of the Public Health Act; this is the Public Health (Prevention of COVID-19) (Requirements and Conditions of Entry into Uganda) Order [24]. The Order categorized travelers according to risk in the country of origin and the process of handling them after testing for COVID-19. The Order introduced additional health measures like institutional quarantine or isolation of travelers.

### Prohibition of entry into Uganda

On 24th March 2020, the Minister enacted the Public Health (Prohibition of Entry into Uganda) Order [25]. This Order closed the borders of Uganda except for persons working with the United Nations, other humanitarian organizations, cargo vehicles and aircraft. The Order also restricted the numbers of persons entering Uganda and gave powers to the medical officer of health to examine any cargo vehicle, vessel, or aircraft and any persons on board. The Minister amended this Order five times to provide for the number of crew on-board of cargo vehicles, vessels and aircraft; to continuously extend the closure of the borders and to prohibit entry of some travelers until when borders were opened.

### Other legislative provisions applied to the COVID-19 response

Other than the Rules under the Public Health Act Cap 281, we applied the UPDF Act 2005 [15]. Due to the nature of the pandemic, there was a national security concern that necessitated the involvement of the Army under Sect. 42 of the UPDF Act. This section empowers the Army to appoint its officers to work with any part of the Government to control any event that is likely to cause a national disturbance. Therefore, the Army was involved in the enforcement of the COVID-19 Rules at the international borders and in the appointment of the COVID-19 Incident Commander. Similarly, the COVID-19 outbreak happened in the middle of the financial year 2019/2020, hence, the Ministry of Health had to get supplementary funding under Sect. 25 of the Public Finance Management Act, 2015 (PFMA) [14]. Characterization of COVID-19 as a disaster required multi-ministerial action thus the application of the UPDF Act and PFMA to control the outbreak.

Additionally, the national policy for Disaster Preparedness and Management, 2010 [16] of the Office of the Prime Minister was implemented. The Policy provides for the way government should cooperate in the case of a pandemic that spreads very fast due to increased global travel. Again, the policy lists the Ministry of Health; the Office of the Prime Minister (Disaster Management); Ministry of Internal Affairs (Immigration and Policy); Ministry of Defense (Uganda Peoples’ Defense Forces); Ministry of Information and National Guidance; Ministry of Local Government and District Local Governments as the institutions that partnered to control the pandemic.

### Court cases arising from COVID-19 preventive measures

Due to the restriction and controls under the COVID-19 Rules, there were cases filed against the Attorney General during the period covered by this paper. The Public Health (Control of COVID-19) Rules imposed the restrictions on cinemas, theaters, business premises, places of worship, political rallies, and so on. The case of *Theatrical Association of Uganda and Another versus the Attorney General* (Misc. Cause 369 of 2021) challenged the closure of entertainment centers and open-air concerts. In addition, the case of *Kiganda Michael versus the Attorney General* (Constitutional Petition No. 20 of 2021) contested the closure of places of worship or prayer. Again, the case of *Tumuheirwe Arthur versus the Attorney General* (Misc. Cause No. 382 of 2020) in which the closure of business premises was challenged. Equally, the case of *Mgugu Abbey versus Electoral Commission and the Attorney General* (Misc. Cause No. 63 of 2020) related to the restrictions on political activities. Following the Public Health (Control of COVID-19) (No. 2) Rules, the case of *Turyamusiima Geoffrey versus the Attorney General and Dr. Jane Ruth Aceng* contested the failure of listing legal services among the essential services in Rule 8 [26]. This led to the amendment of the same law through statutory instrument No. 64 of 2020 to include legal services with some restrictions. Additionally, the case of *Male H. Mabirizi versus Attorney General* (Misc. Cause No. 193 of 2021) challenged the implementation of presidential speeches without enactment of any Act of Parliament or statutory instruments.

### Timelines of the rules, preventive measures and outbreak progress

**Fig. 2:**
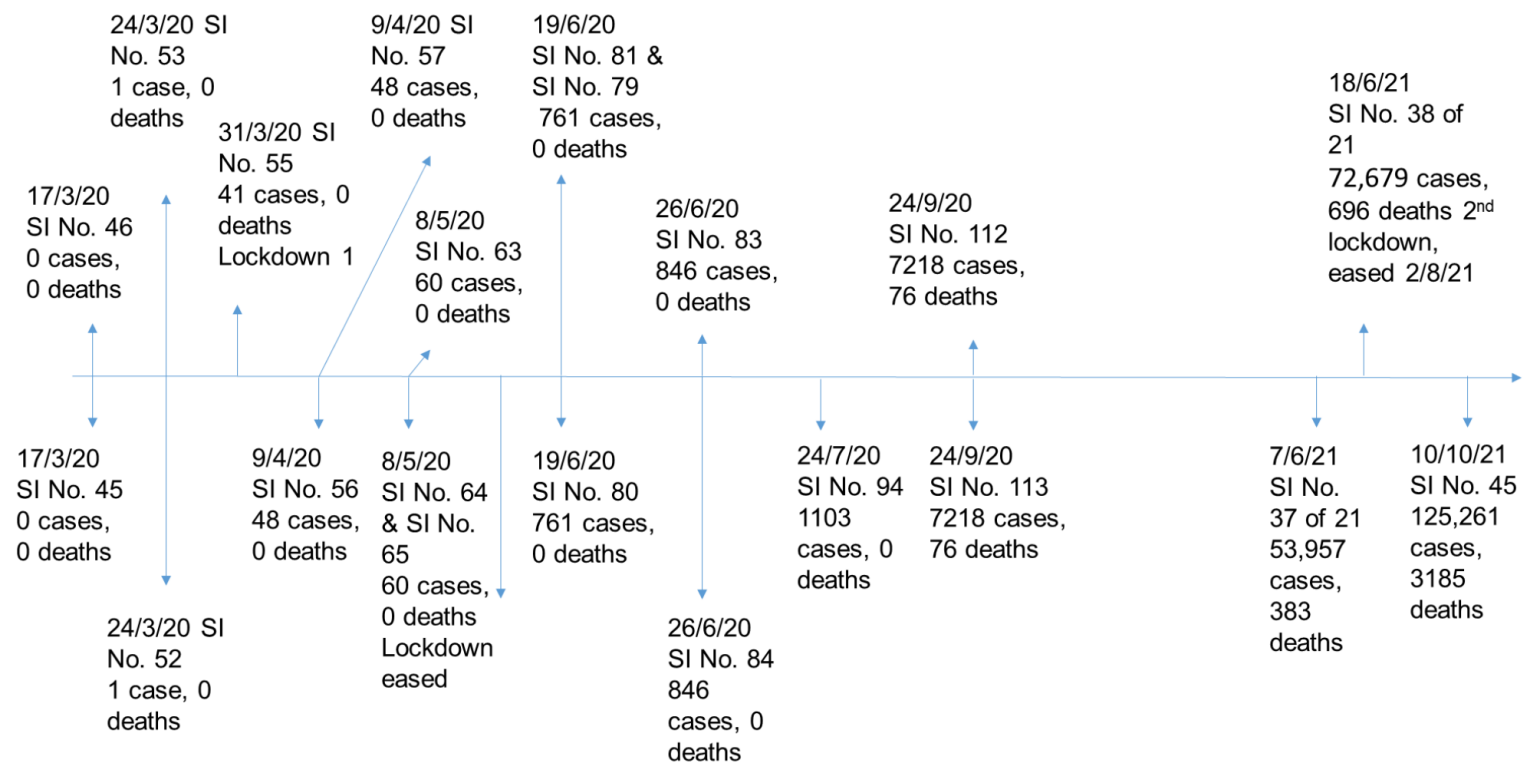
Timeline showing Rules developed aligned to outbreak progress. This figure (not to scale) presents the date, number of cases, deaths and how the Rules and Orders were made over time. The Statutory Instrument (SI) number shows the sequence of passing of the Rules or Orders. Although the cases and deaths continued increasing in number, the legislation changed with the outbreak severity

The dates of enactment of the Rules, the Statutory Instrument number, and the number of cases and deaths recorded are shown in Fig. 2 (not to scale). The figure depicts the relationship between the public health measures (NPIs) and the spread of COVID-19 over time in Uganda. Noteworthy, security agencies supported enforcement of the public health measures although discrimination and violence increased in Uganda [27].

## Discussion

This study highlights five key findings. First, Presidential speeches were an effective medium for delivering COVID-19 public health messages (Non-pharmaceutical Interventions) to the public. However, these pronouncements and directives were not law in Uganda thus needed enactment of public health Rules to support enforcement. Secondly, the declaration of COVID-19 under the Public Health Act was key to implementing the NPIs. Third, the enactment of COVID-19 Rules was necessary but not sufficient to control the pandemic, hence other laws came into play. Fourth, the NPIs evolved along the pandemic curve and there was a need to realign the COVID-19 Rules frequently as appropriate. Finally, enacting COVID-19 Rules attracted specific litigation and somewhat infringed on certain human rights provisions like right to work when the business premises were closed and the right to practice one’s faith when the places of prayer were closed.

Uganda was among the first countries in sub-Saharan Africa to enact COVID-19 specific laws as early as 17th March 2020. Presidential speeches were an effective medium for delivering COVID-19 public health measures (Non-Pharmaceutical Interventions) to the public that relied on sound scientific evidence such as recommendations from the World Health Organization and Centers for Disease Prevention. However, these pronouncements were not law in Uganda thus, the need to enact public health Rules for enforcement. Article 23 (1) (d) of the 1995 Constitution of Uganda provides for the withdrawal of personal liberties for the purpose of preventing spread of an infectious or contagious disease. Hypothetically, if the Public Health Act (Cap 281) did not exist the 1995 Constitution would be sufficient. The last time Uganda applied Sect. 27 of the Public Health Act, namely, the Public Health Rules (Plague Control), Statutory Instrument 281–27 was in the 1980s. Recently, Uganda has successfully contained several highly contagious disease outbreaks like Cholera, Yellow Fever, Ebola Virus Disease [7, 28, 29], without necessarily enacting special laws. What was unique with COVID-19, however, was the high-level political commitment with Presidential speeches legalized by enacting Rules.

Like Uganda, other sub-Saharan countries such as South Africa [30], Kenya [31], and Botswana [32] in late March and April 2021 applied laws to respond to the COVID-19 outbreak. Higher-income countries including China in Asia [33], New Zealand in Oceania [34], the United Kingdom in Europe [35], and the USA in the Americas [36] did the same. While some countries issued subsidiary legislation under their public health principal law, others amended related laws. For instance, in April 2020, Kenya passed the Public Order (State Curfew) Variation Order to enforce a curfew [37]. In June 2020, Trinidad and Tobago passed the Public Health [2019 Novel Coronavirus (2019-nCov)] (No.12) Regulations, 2020 under the Public Health Ordinance, Ch. 12 No. 4 [38]. The Rules imposed restrictions on public gatherings, hours of business operations, casinos, cinemas, theatres, gyms, school establishments, and restaurants. The same Rules provided guidelines on quarantine, treatment of COVID-19 patients, closed Trinidad borders, and prohibited testing in private medical laboratories. In China, laws were used to enforce compliance and control the spread of COVID-19. The Chinese government used experts to make COVID-19 specific laws under the Chinese Criminal law [33] that made it an offense for a person to violate laws on the prevention and control measures against COVID-19.

Notably, the NPIs evolved along the pandemic curve and there was a need to realign the COVID-19 Rules as frequently as appropriate. The COVID-19 Rules were dynamic with “sunset provisions” of expiry. These frequent amendments (21 in total) of the COVID-19 Rules defined the principle of predictability of laws, “Stare decisis”. This observation was consistent in South Africa which used the Disaster Management Act 2002 to develop, implement and amend several core Rules depending on the five levels of outbreak alertness [39]. Although the provisions in the Public Health Act 1984 (Control of Disease) were seemingly adequate at the start [40], the United Kingdom enacted a new Coronavirus Disease Act, 2020 [41] that was reviewed often to add, remove or renew provisions as an outbreak evolved.

Some of the COVID-19 preventive health measures (NPIs) directly restrained human rights such as the right to education (Article 30), the right to practice any religion (Article 29), and the right to move freely throughout (Article 29), to enter, leave and return to Uganda. This scenario was prevalent globally and not in Uganda alone [42]. Noteworthy, these COVID-19 preventive measures became more stringent as the outbreak evolved with increasing cases and deaths, such as community quarantine or total “lockdown” [43]. Perhaps, it is the perception of infringement of human rights that triggered litigation in courts of law in Uganda, Kenya [43, 44], Southern Africa [45], the UK [46], and the USA [47]. Overall, the decisions of the court upheld the provisions of COVID-19 Rules across different jurisdictions. The cases filed in Ugandan courts challenged the implementation of the presidential pronouncements, some of which received judgment by the time of this study. In Uganda, while the Courts upheld the Rules in the case filed to permit political rallies [48]; they favored the case of reclassifying the Legal fraternity as essential workers. In the United States of America, the courts at the beginning of the pandemic rejected challenges to COVID-19 emergency orders but later on supported them [49]. It is recommended to have an active review of the role of policies in evidence-based decision-making during outbreaks [50].

The findings of this study suggest several practice and policy considerations. Certainly, the legal fraternity has a role to play in the control of pandemics. However, the available legal expertise in Uganda, for example, the Health Cluster of the Uganda Law Society, was limited to medical laws and not necessarily the wider public health realm. Although public health interventions may be lifesaving, these should be enforced through laws while respecting human rights. The plethora of legal suits arising from the general provisions and specific Rules to control COVID-19 in Uganda reinforces this argument.

This study delineates key information gaps for future research. Looking back, whether enactment of specific Rules to contain pandemics such as COVID-19 is relevant requires further study. Further, the successful control of previous outbreaks such as Ebola in Uganda was done without enacting specific Rules, which lends credence to this hypothesis. Additional studies would investigate the impact of enacting COVID-19 specific Rules concerning human rights and other unforeseen undesirable effects in Uganda. For example, religious freedoms were curtailed and delays in accessing primary health care for HIV/AIDS or pregnant women during enforcement of NPIs were reported. A crucial aspect of research would be when and how to integrate scientific evidence, public health interventions, and legal provisions. For example, what NPIs go into law and what goes into SOPs would be informative and possibly efficient given the evolving scientific evidence base in previous unknown pandemics such as COVID-19. Finally, it would be informative to understand the interaction of local COVID-19 Rules and the WHO International Health Regulations 2005 for cross-border health.

Shortcomings of this study included limited consultation with a broader scope of stakeholders for ‘buy-in’, due to the unique emergency response circumstances caused by the COVID-19 pandemic. However, this was overcome by Presidential speeches with directives informed by the COVID-19 National Task Force at the Office of the Prime minister, the sector-specific COVID-19 task force at the Ministry of Health, the Scientific Advisory Committee, and other lobbyists such as school owners and operators. Still, this study had a key strengths in employing a legal perspective in documenting the Non Pharmaceutical Interventions for the COVID-19 outbreak in Uganda and applying multiple sources of data to corroborate the findings such as Presidential speeches, resolutions of Cabinet meetings, Statutory Instruments, the registry of court cases in Uganda and COVID-19 situation reports;

In conclusion, it was possible to successfully enact laws in Uganda specifically to control the spread of COVID-19 and possibly mitigate its impact. There were challenges faced beyond the court cases, for example, the COVID-19 response was interpreted variously during the political election campaign; cross border collaboration was limited; controlling COVID-19 outbreak in prisons and unwanted effects of the interventions (NPIs). The perceived infringements on individual human rights need to be balanced with the quest for community survival as enshrined in the Constitution of the Republic of Uganda. As such, the study recommends public sensitization about legislative provisions and reforms to guide public health responses in future outbreaks or pandemics. Finally, countries of similar settings could consider a platform to share “best practices” in enacting Rules for controlling disease outbreaks.

## Data Availability

The data that support the findings of this study including the published Rules are available from *The Uganda Gazette*. Some court cases are not finalized thus, restrictions apply to the availability of some data (court files, proceedings) and so are not publicly accessible. These Rules (Statutory Instruments), other accompanying laws are publicly available in the Uganda Gazette and the Uganda Legal Information Institute: https://ulii.org/. Other data sources such as Presidential speeches are also available on Uganda Media Centre: https://www.mediacentre.go.ug/, https://covid19.gou.go.ug/uploads/document_repository/authors/h.e._yoweri_kaguta_museveni/Address_by_H_E__Yoweri_Kaguta_Museveni_on_Corona_Virus_18th_March_2020.pdf; https://www.health.go.ug/covid/document/presidential-address-on-corona-virus/. Some of the datasets generated and analyzed during the current study specifically the court cases are not yet publicly available (until cases are disposed of) online except in physical form but are available from the corresponding author on reasonable request.
